# In Response to Comments Made in “S100B Protein and Chronic Subdural Hematoma”

**DOI:** 10.3389/fneur.2013.00026

**Published:** 2013-03-15

**Authors:** Eric Peter Thelin, Malin Elisabet Persson, Bo-Michael Bellander

**Affiliations:** ^1^Department of Clinical Neuroscience, Section for Neurosurgery, Karolinska InstitutetStockholm, Sweden; ^2^School of Medicine, The University of ManchesterManchester, UK

We recently published the paper; “Case Report: Extreme Levels of Serum S100B in a Patient with Chronic Subdural Hematoma” (Persson et al., 2012), in Frontiers in Neurology. It was a case report concerning a patient with a subdural mass, neurological deterioration, and unusually high S100B levels in serum. Further clinical examination proved that the patient was suffering from a malignant melanoma.

We fully agree with Drs Gelabert-González, Aran-Echabe, and Serramito-Garcíain their manuscript “S-100 B protein and chronic subdural hematoma” that patients presenting chronic subdural hematomas do not normally benefit from biomarker (S100B and NSE) monitoring (Gelabert-González et al., 2013).

In the present case the patient was admitted to the neurosurgical intensive care unit unconscious, GCS 3, and presenting a dilated pupil. The admission CT showed a subdural mass with severe midline shift.

The biomarker S100B is used frequently in our NICU to monitor comatose patients and in this case the level was significantly increased. Although the CT findings indicated that the patient was suffering from a chronic subdural hematoma, it later on was found to harbor metastatic growth from an initially unknown recurrence of a malignant melanoma.

Patients suffering from malignant melanomas might present “false” high S100B levels after traumatic brain injury which has to be considered when there seems to be a discrepancy between clinical findings and the serum level of S100B.

The title was chosen because of the initial confusion concerning CT findings and neurology, and is kept to illustrate the complexity of this case.

In the article, as is proposed by the authors Gelabert-González, Aran-Echabe, and Serramito-García, we never suggest that S100B is an important marker for chronic subdural hematoma. Instead we highlight the potential influence of other, non-glial sources of S100B that could affect diagnosis and clinical decision-making of patients in the neurointensive care unit.
